# Timosaponin-BII inhibits the up-regulation of BACE1 induced by Ferric Chloride in rat retina

**DOI:** 10.1186/1472-6882-12-189

**Published:** 2012-10-22

**Authors:** Ju-Fang Huang, Lei Shang, Pei Liu, Meng-Qi Zhang, Shuang Chen, Dan Chen, Chun-Ling Fan, Hui Wang, Kun Xiong

**Affiliations:** 1 Department of Anatomy and Neurobiology, School of Basic Medical Sciences, Central South University, Changsha, Hunan, 410013, China; 2 Five-year Medicine Program, Grade 2009, Central South University Xiangya School of Medicine, Changsha, Hunan, 410013, China; 3 Eight-year Clinical Medicine Doctor Program, Grade 2006, Central South University Xiangya School of Medicine, Changsha, Hunan, 410013, China

**Keywords:** Timosaponin-BII, Oxidative stress, BACE1, Retina, Aβ

## Abstract

**Background:**

Our previous studies indicated that oxidative stress up-regulated the expression of β-amyloid precursor protein cleavage enzyme-1 (BACE1) in rat retina. Pharmacological reports have shown Timosaponin-BII, a purified extract originating from Chinese medical herb *Rhizoma Anemarrhenae,* is characterized as an antioxidant. Our present study aimed to determine whether Timosaponin-BII affected the expression of BACE1, β-amyloid precursor protein cleavage production of Aβ1-40 and β-C-terminal fragment (β-CTF) in rat retina, which were pre-treated with the oxidizing agent (solution of FeCl_3_).

**Results:**

Few distinctions of BACE1 distribution were observed among all groups (normal control group, model group, Timosaponin-BII treated and vehicle control groups). Rat retinas in model group and vehicle control group manifested an apparent up-regulation of BACE1 expression. Meanwhile, the level of malonaldehyde (MDA), Aβ1-40 and β-CTF were increased. However, when comparing with the vehicle control group, the retinas in Timosaponin-BII treated group showed significantly less BACE1 (*p*<0.05) and accumulated less Aβ1-40 or β-CTF (*p*<0.05). It also showed significantly decreased level of MDA (*p*<0.05) and prolonged partial thromboplastin time (*p*<0.05).

**Conclusion:**

Our data suggested that Timosaponin-BII remarkably inhibited the up-regulation of BACE1 and reduced the over-production of β-CTF and Aβ in rat retina, which was induced by FeCl_3_. The mechanism of Timosaponin-BII on BACE1 expression may be related to its antioxidant property.

## Background

Alzheimer’s disease (AD) is a progressive neurodegenerative disease prevalent in aged populations and characterized by the presence of senile plaques (SP), neurofibrillary tangles (NTFs), capillary cerebral amyloid angiopathy (capCAA) and the abnormal loss of synapses [1,2]. The amyloid deposition, whose disposal mechanisms have attracted great attention to neurologists, is regarded as a main etiology. It has been documented that accumulation of amyloid β-peptide (Aβ), which is a cleavage form of β-site amyloid precursor protein (β-APP), acts as a fundamental initiator of this disease [3]. β-APP is cleaved by β-amyloid precursor protein cleavage enzyme-1 (BACE1), along with the digestion by γ-secretase, which results in the release of Aβ [4]. Therefore, BACE1 plays a significant role in secretion of Aβ. Previous studies have shown that different pathological conditions, including traumatic brain injury, cerebral ischemia, oxidative stress, functional deprivation, lead exposure, ischemia and hypoxia, contribute to the up-regulation of BACE1 expression or facilitate its activity, and eventually lead to the massive accumulation of Aβ [5-11]. Our previous data showed that oxidative stress induced by the intra-vitreous injection of FeCl_3_ solution elevated the expression of BACE1 in rats’ retina, and consequently triggered the dramatic production of Aβ [12]. This finding is in agreement with other studies [13-15]. It is generally accepted that up-regulation of BACE1 expression as well as the degree of activated BACE1 is closely related to the aggravation and the onset of AD [16,17]. Therefore, investigations focused on inhibiting either excessive generation or hyperactivity of BACE1 are extensively established on account of alleviating the symptoms or even eradicating of the disease in early stage.

Zhimu, a traditional Chinese medicine, is derived from the dry rhizome of the plant *Anemarrhena asphodeloides Bge*, Numerous steroids have been extracted from this plant, such as *sarsasapogenin*, *markosapogenin*, *negitogenin*, and their glycosylated products named Timosaponin-AI, -AII, -AIII, -AIV, -BI and -BII [18]. It has been reported that Timosaponin-BII has a neuronal protective and anti-inflammatory effect possibly by suppressing the production of pro-inflammatory factors IL-1, IL-6 and TNF-α [19-21]. The dementia-palliative effect of Timosaponin-BII may involve multiple mechanisms and one of them is its potential anti-oxidative property. An intriguing observation from Ouyang’s experiment showed that Timosaponin-BII diminished the Aβ-induced oxidative impairment by promoting scavenging of superoxide radicals [22]. Nevertheless, it is to be elucidated whether this neuronal protective effect would inhibit the oxidant-induced up-regulation of BACE1 expression and even would attenuate the overproduction of Aβ with its anti-oxidant property. In the present study, we constructed an oxidative impairment animal model by intra-vitreous injection of FeCl_3_ solutions then administered Timosaponin-BII to these animal models. The current study aimed to determine whether Timosaponin-BII could inhibit the production of BACE1 in the retina including Aβ and malondialdehyde (MDA), which is a lipid oxidative product.

## Methods

### Reagents and Animals

#### Reagents

The powder of Timosaponin-BII was provided by Professor Wan-Sheng Chen from Department of Pharmacology, School of Pharmacology, Second Military Medical University, Shanghai, China [23] and its purity is above 98%. Timosaponin-BII was dissolved in normal saline (NS) at 1.2 g/ml in room temperature (24°C), subsequently diluted FeCl_3_ (Sigma, MO, USA) with 0.01M PBS at 10 mM to yield a low concentration working solutions. The working concentration to induce the proper increase of BACE1 expression and Aβ aggregation has been determined in our previous study [12].

#### Animals

Twenty-four adult male SD rats weighing 200–250 g and aged 2–3 months were purchased from Animal Center of Xiangya School of Medicine, Central South University. All rats were isocoria with eyes observed transparent and no deformity. The laboratory animals were housed in a constant temperature at 24°C with relative humidity at (55±10) %. All experiments were carried out according to the principles outlined in the NIH Guide for Care and Use of Laboratory Animals. The present study was approved by Animal Ethics Committee of Xiangya School of Medicine, Central South University.

### Animal model construction and drug administration

Rats were equally divided into four groups randomly: The normal control group (CTL), the model group (FeCl_3_), the vehicle control group (FeCl_3_+NS) and the experimental group (FeCl_3_+Timosaponin-BII). The methods of constructing models were previously described in detail in our paper [12], and briefly described as follows: We anaesthetized the rats with chloral hydrate by peritoneal injection and fixed them at stereotaxic apparatus with eyes properly exposed. Afterwards, we extracted equal amount of humor vitreous and injected the FeCl_3_ solution at 5 μl with a needle penetrating the eyeball from the dorsal sclera at 1 mm posterior to the cornea–sclera junction and toward the center of the eye for 2 mm deep. The procedure was cautiously carried out to avoid penetrating the beyond lens. The experimental group received Timosaponin-BII at 6 mg/kg by intravenous injection every morning for 14 days [24] while the vehicle control group was given an injection of equal amount of normal saline. No drugs were administered to the normal control or model group. At the 14^th^ day, we randomly selected three in each group for morphological studies while the remaining animals were used for biochemistry studies.

### Prothrombin time assay

Blood samplings were collected by puncturing the heart using natrium citricum negative pressure tubes that was provided by Department of Laboratory, the Third Xiangya Hospital of Central South University. The rats were deeply anaesthetized with chloral hydrate and their chests were promptly opened in order to expose the heart. Consequently we punctured the left artrium with a negative pressure tube. Three milliliters of blood samples were collected from the experimental group and the vehicle control group for the determination of Prothrombin time (PT), Activated partial thromboplastin time (APTT), Thrombin time (TT) and the concentration of Fibrinogen (FIB). Subsequently, the rats were transcardiacally perfused.

### Immunofluorescence staining

The eyeballs were removed following trans-cardiac perfusion with 4% paraformaldehyde in PBS at certain survival time point, post-fixed in the perfusion solution overnight, followed by cryo-protection in 30% sucrose. The eyeballs were cut in a cryostat at 14 μm thickness. Cross-retinal sections near and passing the optic nerve head were thaw-mounted on Superfrost Plus slides (VWR, PA, USA).

Sections were incubated overnight at 4°C either with BACE1 antibody (1:200; polyclone, AB5940, Millipore, MA, USA) after blocked by PBS solution containing 10% BSA and 0.3% Triton X-100 (Sigma, MO, USA) for 1 hr. Immunoreaction products were visualized following 2 hr incubation with Alexa Fluor® 568 conjugated donkey anti-rabbit IgG (1:200; Invitrogen, CA, USA). Sections were counterstained with Hoechst 33342 (1:50k; Sigma, MO, USA), washed, and mounted with anti-fading medium before microscopic examination. Digital images were obtained using fluorescence microscopy (Olympus, BH-40, Tokyo, Japan).

### Western blotting

For biochemical studies, the eyeballs were removed following a vascular rinse with ice cold PBS under overdose anesthesia (sodium pentobarbital 100 mg/kg, i.p.) in each group. The retinas were dissected out in cold PBS on ice, carefully soaked with a piece of filter paper, then collected in 1.5 ml test tubes and weighed. The retinas were homogenized by sonication in cell lysis buffer (10× w/v, Pierce Inc., CA, USA) containing a cocktail of protease inhibitors (Roche, IN, USA). Tissue lysates were centrifuged (Ependoff, CA, USA) at 15,000×*g* at 4°C for 15 min after standing on ice for 1 hr. Supernatants were collected and protein concentrations were determined by BCA protein assay (Pierce Inc., CA, USA). Equal quantity of protein (40–100 μg depending on target proteins) was run on 10% SDS-PAGE gel (Bio-Rad, CA, USA) after protein denaturation. The polypeptides were electro-transferred to Trans-Blot® pure nitrocellulose membrane (Bio-Rad, CA, USA). Non-specific binding was blocked with PBS containing 5% non-fat milk. Nitrocellulose membranes were incubated with primary antibodies to BACE1, APP (1:500; polyclone, AHP538, Serotec, CA, USA) and β-tubulin (1:10k; polyclone, Sigma, MO, USA) overnight in 4°C. Membranes were further incubated in HRP-conjugated secondary antibodies (1:20k; Bio-Rad, CA, USA) for 1 hr. Protein bands were visualized with an ECL Plus™ Western Blotting Detection kit according to manufacturer’s instruction (GE Healthcare Life Sci., NJ, USA).

### Aβ1-40 ELISA Assay

Aβ 1–40 levels in retina extracts were assayed using a commercial kit according to manufacturer’s instruction (Biosource International, CA, USA), except that the reporter antibody supplied by the manufacturer was replaced with biotinylated 4G8 at 1:4k (Signet Laboratories Inc., MA, USA). Equal quantity (80 μg) of protein was loaded in each well and each analysis performed in duplicate.

### MDA concentration assay

MDA levels in retina extracts were assayed using a commercial kit according to manufacturer’s instruction (Jian-Cheng Biotechnical Co., Nanjing, China), the standard reference substance named tetraethoxypropane were used in 10 nmol/ml. Equal quantity (100 μg) of protein was loaded in each well and each analysis performed in duplicate.

### Statistic analysis

Specific density was calculated by subtracting background density on the membrane from total density measured over the protein band, followed by standardization to β-tubulin references using the software of Image J (Image J, MD, USA). For ELISA data, levels of Aβ1-40 were determined by a standard curve generated using serially diluted synthetic Aβ1-40 peptide provided in the kit. Finally, all data were normalized to the means of inner reference (for western blotting) or the means of control group (for ELISA data) whenever appropriate, yielding relative values expressed as percentages of controls (control serve as 100%).

Statistical analyze of normalized values were conducted using one-way ANOVA followed by PASW SPSS 19.0 (SPSS, CA, USA), yielding *p* values between individual groups. The data expressed as means±SD. The minimal significant level of difference between groups was set at *p*<0.05.

Digital images of retinal immunolabeling were captured at 40× objectives at comparable retinal segments (~0.5 mm apart from the optic head). All illustrations were prepared with Adobe Photoshop CS 6.0 (Adobe, CA, USA). Whole panel images were converted to TIFF format, and contrast/brightness was adjusted if necessary.

## Results

### Timosaponin-BII prolonged APTT

**Figure 1:**
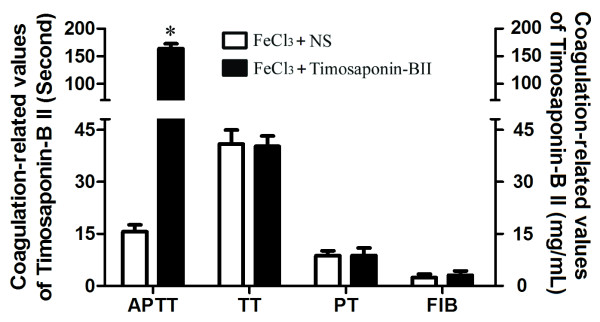
**Effect of Timosaponin-BII on Prothrombin time (PT).** Activated partial thromboplastin time (APTT), Thrombin time (TT) and the concentration of Fibrinogen (FIB). *: compared with vehicle control group: *p*<0.05, n=3.

When compared with the vehicle control, Timosaponin-BII treated rats had no difference in TT, PT and the concentration of FIB while the APTT was significantly longer (Figure 1). This result indicated that Timosaponin-BII had an inhibitory effect on intrinsic coagulation system, which is in agreement with a previous study of Lu et al. (2011) [24]. 

### Timosaponin-BII decreased BACE-1 expression in rat retina

**Figure 2:**
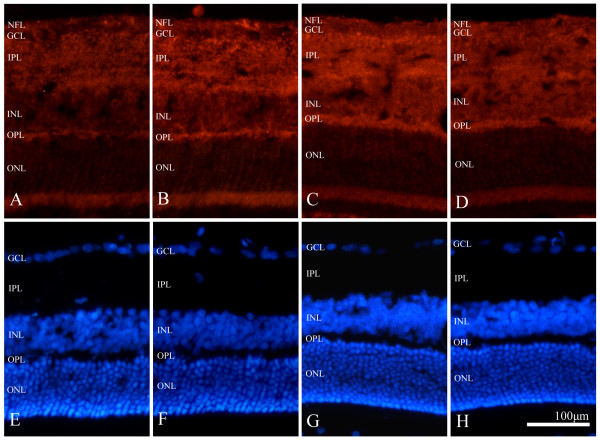
**Immunofluorescence staining of BACE1 in rat retina.A-D** illustrated the laminar-specific BACE1 expression in normal group (**A**), model group (**B**), vehicle control group (**C**) and experimental group (**D**). E-H illustrated Hoechst staining in normal rat (**E**), model group (**F**), vehicle control group (**F**) and experimental group (**H**). Scale bar =100 μm in H applied to all panels.

The immunofluorescence staining result showed that BACE1 was mainly present in inner plexiform layer (IPL), outer plexiform layer (OPL) and the nerve fiber layer (NFL). Slight cellular dispersion also existed in the inner and out nuclear layers. These results were consistent with our previous studies [12]. In contrast with the normal control, the FeCl_3_-treated animal model showed significantly more distinct and heavier BACE1 immunoreactivity and exhibited no apparent discrepancies in the cellular and laminar distribution of BACE1. The vehicle control showed a similar expression pattern as the model group. Although no difference in distribution was detected, the experimental group displayed remarkably lower immunoreactivity when compared with the model group and the vehicle control, but it remained higher than the normal control (Figure 2). 

**Figure 3:**
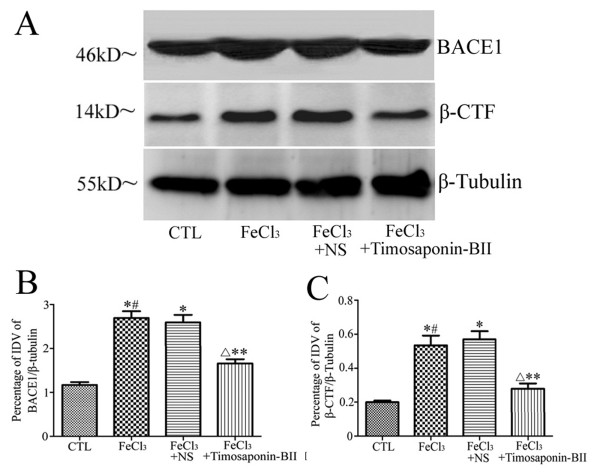
**Western blotting analysis result of BACE1 and β-CTF. A**: bands of BACE1, β-CTF and β-tubulin; **B**: optical density analysis of BACE1; **C**: optical density analysis of β-CTF. *: compared with normal control group: *p*<0.05; #: compared with vehicle control group: *p*>0.05; Δ: compared with normal control group: *p*<0.05; **: compared with model groups or vehicle control group: *p*<0.05, n=3.

The western blotting results showed that BACE1 was mainly exhibited as a single 46 kDa band in all groups. The bands in model group as well as in the vehicle control group were apparently thicker and larger than those of normal control group. The band in the experimental group was thinner and smaller than those in model group and vehicle control but remained larger than the normal ones (Figure 3A).

### Timosaponin-BII decreased the overproduction of β-CTF in rat retina

β-CTF (BACE1 cleavage product) and β-tubulin were mainly exhibited as a single 14 kDa band or 55 kDa band respectively in all the four groups. The bands of β-CTF in model group as well as the vehicle control group were apparently thicker and larger than normal control. The bands in the experimental group were thinner and smaller than those in model group and vehicle control but remained larger than the normal ones (Figure 3A).

The optical density measurement and statistical analysis indicated that the vitreous injection of FeCl_3_ up-regulated the expression of BACE1 and resulted in the overproduction of β-CTF. The vehicle control group showed a similar result with the model group. The rats that were administered with Timosaponin-BII apparently manifested a significant decrease in BACE1 expression and β-CTF production in relation to the model group and the vehicle control groups but still remained higher than the normal ones (Figure 3B, C).

### Timosaponin-BII inhibited overproduction of Aβ in rat retina

**Figure 4:**
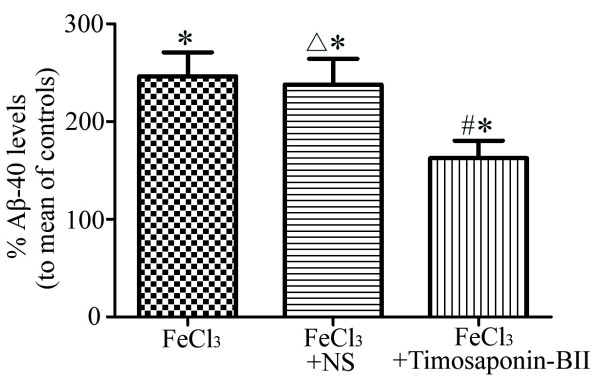
**Statistic analysis of Aβ1-40 level in rat retina in relation to the normal control.** *: compared with normal control group: *p*<0.05; △: compared with model groups: *p*>0.05; #: compared with model group or vehicle control group: *p*<0.05, n=3, control serve as 100%.

The statistics analysis of ELISA result for Aβ1-40 level was described in Figure 4. No discrepancies in Aβ1-40 concentration were observed between the model group and the vehicle control group but both the two groups showed the upsurge in relation to the normal control group. The level of Aβ1-40 in Timosaponin-BII treated group was lower than those in model group and vehicle group but remained higher than those in the normal control group. This result indicated that Timosaponin-BII remarkably inhibited overproduction of Aβ that was induced by up-regulation of BACE1. Nevertheless, the effect of Timosaponin-BII was limited in extent since the level of target peptides was still higher than those in normal condition.

### Timosaponin-BII reduced MDA production in rat retina

**Figure 5:**
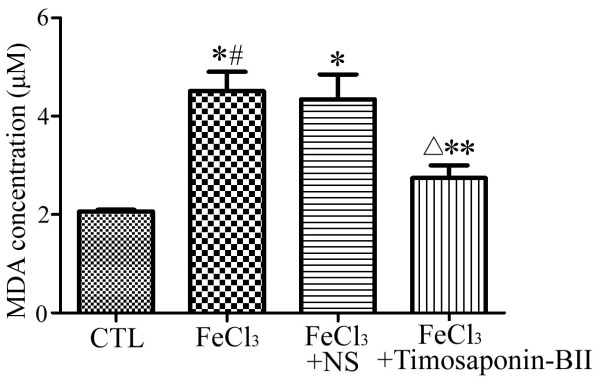
**Statistical analysis of MDA level in rat retina.** *: compared with normal control group: *p*<0.05; #: compared with vehicle control group: *p*>0.05; △: compared with normal control group: *p*<0.05; **: compared with model group or experimental group: p<0.05. n=3.

The statistics analysis of MDA level is shown in Figure 5. No discrepancies in MDA concentration were observed between the model group and the vehicle control group but both groups showed the upsurge in relation to the normal control group. The level of MDA in the experimental group was lower than those in model group and vehicle control group but remained higher than those in the normal control group. This result indicated that Timopaponin-BII reduced the oxidative stress that was induced by the FeCl_3_ in rats’ retina. Nonetheless, the capacity was limited as the oxidative stress remained higher in the normal control group.

## Discussion

Alzheimer’s disease (AD) is a progressive neurodegenerative disease, which is characterized as deterioration of memory and global cognitive ability, in addition to the impairment of language skills and the extreme apathy and exhaustion [25,26]. Senile plaques, neurofibrillary tangles, capillary cerebral amyloid angiopathy and the abnormal loss of synapses are proposed to be the principal pathological changes in AD [1,2]. Moreover, the amyloid plaque originating from the β-APP abnormal proteolysis product Aβ is considered to be the predominant feature of the disease. In addition to the frontal lobe, occipital lobe and the hippocampus that were traditionally identified with pathological molecular abnormalities in AD, retina has recently been documented to be equivalently involved in the morbidity process [27]. Some specialists point out that the detectable Aβ would turn up in patients’ retina in the early stages rather than advanced ones, which provides pre-diagnostic evidence before the occurrence of dementia [28]. The pertinent experiment carried out on animals also reaches a similar conclusion. Over-production of Aβ and β-amyloid plaques in retina were detected prior to behavior transformation and cognitive impairment in AD-transgenic animals [28,29]. Dependent on aforementioned facts and data, we selected retina as an experimental object to explore the effect of Timosaponin-BII on Aβ laid in this conclusion and relevant evidence about Aβ presence inferred from our previous studies [11,12].

Oxidative stress has long been unequivocally esteemed as one of the principal etiological factors of AD [30-33]. Related reports illustrated that Aβ had an oxidative toxicity for neurons [34], while current evidence supports the amplifying effect of oxidative stress on Aβ aggregation [7]. Thus, a complete self-enforcing circle including Aβ accumulation and reactive oxygen production eventually resulted in neuronal death. Our previous data revealed that the FeCl_3_ induced oxidative stress up-regulated BACE1 expression [12], compatible with Tamagno’s conclusion that oxidative stress promoted BACE1 activation in NT2 cells [15]. In our present study, the apparent increase of BACE1 protein and cleavage product Aβ, as well as MDA in FeCl_3_ treated retina indicated the success in constructing the oxidative model.

Timosaponin-BII is widely accepted as neuronal protective chemicals extracted from Chinese traditional herb (*Anemarrhena asphodeloides*). Nevertheless, the mechanisms of its prevention for neurons from injury still remain to be further elucidated. Several primary hypotheses contribute to this issue. First of all, Timosaponin-BII may inhibit the inflammation in nerve system under pathological conditions in the context of its inhibitory effect on the generation of pro-inflammatory factors IL-1, IL-6 and TNF [20,21,35]. Second, Timosaponin-BII partly restores rats’ intelligence quality that is suppressed by Aβ aggregation through the ameliorating activity of acetylcholine esterase (AchE) [36]. Third, some specialists believe that anti-platelet and antithrombotic activities of Timosaponin-BII potentially prevent the recurrence of thrombus formation and preserve cerebral blood flow, contributing its neuroprotective effect against cerebral ischemia dementia [24,35]. Although little involvement of Timosaponin-BII in extrinsic coagulation system was observed, Lu’s experiment evidently demonstrated its inhibition effect on intrinsic coagulation system. Timosaponin-BII may prevent against the formation and extension of blot clots, which consequently preclude cognitive impairment from ischemia. Our result was in agreement with Lu’s conclusions, but whether the anti-platelet effect influence the blood supply of retina still remains to be investigated. Furthermore, Timosaponin-BII may reduce the direct damage to neurons with its anti-oxidative property [22]. The present data showed that the application of Timosaponin-BII to oxidant injury model led to the decrease of BACE1 protein and Aβ, together with the oxidative product (MDA) *in vivo*. This abatement of BACE1 production associated with the degraded level of MDA in Timosaponin-BII treated animals examined by our group and others. These similar observations may raise conceptual concern to the underlying neuroprotective potential of Timosaponin-BII in oxidative-stress-related aggregation of Aβ in AD pathology. It is tempting to presume that Timosaponin-BII not only prevents the direct attack to neurons from reactive oxygen but also inhibits the process of the “oxidative stress---BACE1---Aβ---neuronal damage” initially with its anti-oxidative capacity, finally resulting in a neuroprotective effect.

It is worthwhile to note with regard to the level of reactive oxygen, BACE1 protein and overproduction of Aβ still remains higher than those of normal control. We believe that the mass of acute and intensive impairment in retina induced with immediate oxidant injection elevates the level of BACE1 protein and Aβ in a short time, while the anti-oxidative capability of Timosaponin-BII prevents further injury caused by reactive oxygen species rather than eliminating the pre-existing pathological molecule, leaving Aβ aggregation in the tissue unvarnished. Additionally, the discrepancy could also be attributed to the complexity in the mechanisms of oxidative stress and the limited competence of Timosaponin-BII as a single monomer from nature plants. At present, there are some limitations of the effects of Timosaponin-BII. However, in terms of its predominant merit in reducing level of oxidant stress and inhibiting the up-regulation of BACE1, our results suggested Timosaponin-BII had the neuroprotective potential through interference with molecules related to Aβ production.

## Conclusion

This present study indicated that Timosaponin-BII inhibited the up-regulation of BACE1 caused by FeCl_3_ in rats retina and reduced the production of Aβ presumably with its anti-oxidant potentials.

## Competing interests

The authors declare that they have no competing interests.

## Authors’ contributions

KX and J-FH designed the experiment. LS, PL and SC performed the experiment. PL and M-QZ drafted the manuscript. HW, C-LF and DC analyzed the data. KX and DC revised the manuscript and participated in paper modification. All authors participated in critical revision of the manuscript and approved the final manuscript.

## Pre-publication history

The pre-publication history for this paper can be accessed here:

http://www.biomedcentral.com/1472-6882/12/189/prepub

## References

[B1] Attems J Yamaguchi H Saido TC Thal DR Capillary CAA and perivascular Abeta-deposition: two distinct features of Alzheimer's disease pathology J Neurol Sci 2010 299 1–2 155 162 20850138 10.1016/j.jns.2010.08.030

[B2] Gotz J Eckert A Matamales M Ittner LM Liu X Modes of Abeta toxicity in Alzheimer's disease Cellular and molecular life sciences: CMLS 2011 68 20 3359 3375 10.1007/s00018-011-0750-2 21706148 PMC3181413

[B3] Selkoe DJ Toward a comprehensive theory for Alzheimer's disease. Hypothesis: Alzheimer's disease is caused by the cerebral accumulation and cytotoxicity of amyloid beta-protein Ann N Y Acad Sci 2000 924 17 25 11193794 10.1111/j.1749-6632.2000.tb05554.x

[B4] Selkoe DJ Alzheimer's disease: genes, proteins, and therapy Physiol Rev 2001 81 2 741 766 11274343 10.1152/physrev.2001.81.2.741

[B5] Blasko I Beer R Bigl M Apelt J Franz G Rudzki D Ransmayr G Kampfl A Schliebs R Experimental traumatic brain injury in rats stimulates the expression, production and activity of Alzheimer's disease beta-secretase (BACE-1) J Neural Transm 2004 111 4 523 536 10.1007/s00702-003-0095-6 15057522

[B6] Wen Y Onyewuchi O Yang S Liu R Simpkins JW Increased beta-secretase activity and expression in rats following transient cerebral ischemia Brain Res 2004 1009 1–2 1 8 15120577 10.1016/j.brainres.2003.09.086

[B7] Tong Y Zhou W Fung V Christensen MA Qing H Sun X Song W Oxidative stress potentiates BACE1 gene expression and Abeta generation J Neural Transm 2005 112 3 455 469 10.1007/s00702-004-0255-3 15614428

[B8] Velliquette RA O'Connor T Vassar R Energy inhibition elevates beta-secretase levels and activity and is potentially amyloidogenic in APP transgenic mice: possible early events in Alzheimer's disease pathogenesis J Neurosci 2005 25 47 10874 10883 10.1523/JNEUROSCI.2350-05.2005 16306400 PMC6725876

[B9] Sun X He G Qing H Zhou W Dobie F Cai F Staufenbiel M Huang LE Song W Hypoxia facilitates Alzheimer's disease pathogenesis by up-regulating BACE1 gene expression Proc Natl Acad Sci U S A 2006 103 49 18727 18732 10.1073/pnas.0606298103 17121991 PMC1693730

[B10] Yan XX Xiong K Luo XG Struble RG Clough RW beta-Secretase expression in normal and functionally deprived rat olfactory bulbs: inverse correlation with oxidative metabolic activity J Comp Neurol 2007 501 1 52 69 10.1002/cne.21239 17206602

[B11] Huang JF Huang K Shang L Wang H Yan XX Xiong K Beta-amyloid precursor protein cleavage enzyme-1 expression in adult rat retinal neurons in the early period after lead exposure Neural Regen Res 2011 6 14 1045 1051

[B12] Xiong K Cai H Luo XG Struble RG Clough RW Yan XX Mitochondrial respiratory inhibition and oxidative stress elevate beta-secretase (BACE1) proteins and activity in vivo in the rat retina Exp Brain Res 2007 181 3 435 446 10.1007/s00221-007-0943-y 17429617

[B13] Misonou H Morishima-Kawashima M Ihara Y Oxidative stress induces intracellular accumulation of amyloid beta-protein (Abeta) in human neuroblastoma cells Biochemistry 2000 39 23 6951 6959 10.1021/bi000169p 10841777

[B14] Paola D Domenicotti C Nitti M Vitali A Borghi R Cottalasso D Zaccheo D Odetti P Strocchi P Marinari UM Oxidative stress induces increase in intracellular amyloid beta-protein production and selective activation of betaI and betaII PKCs in NT2 cells Biochem Biophys Res Commun 2000 268 2 642 646 10.1006/bbrc.2000.2164 10679257

[B15] Tamagno E Bardini P Obbili A Vitali A Borghi R Zaccheo D Pronzato MA Danni O Smith MA Perry G Oxidative stress increases expression and activity of BACE in NT2 neurons Neurobiol Dis 2002 10 3 279 288 10.1006/nbdi.2002.0515 12270690

[B16] Fukumoto H Cheung BS Hyman BT Irizarry MC Beta-secretase protein and activity are increased in the neocortex in Alzheimer disease Arch Neurol 2002 59 9 1381 1389 10.1001/archneur.59.9.1381 12223024

[B17] Li R Lindholm K Yang LB Yue X Citron M Yan R Beach T Sue L Sabbagh M Cai H Amyloid beta peptide load is correlated with increased beta-secretase activity in sporadic Alzheimer's disease patients Proc Natl Acad Sci U S A 2004 101 10 3632 3637 10.1073/pnas.0205689101 14978286 PMC373514

[B18] Ji X Feng YF Advances in studies on saponins in Anemarrhena asphodeloides Chinese Traditional and Herbal Drugs 2010 41 4 S12 S15

[B19] Hu Y Xia Z Sun Q Orsi A Rees D A new approach to the pharmacological regulation of memory: Sarsasapogenin improves memory by elevating the low muscarinic acetylcholine receptor density in brains of memory-deficit rat models Brain Res 2005 1060 1–2 26 39 16226729 10.1016/j.brainres.2005.08.019

[B20] Li TJ Qiu Y Yang PY Rui YC Chen WS Timosaponin B-II improves memory and learning dysfunction induced by cerebral ischemia in rats Neurosci Lett 2007 421 2 147 151 10.1016/j.neulet.2007.04.082 17566650

[B21] Lu WQ Qiu Y Li TJ Tao X Sun LN Chen WS Timosaponin B-II inhibits pro-inflammatory cytokine induction by lipopolysaccharide in BV2 cells Arch Pharm Res 2009 32 9 1301 1308 10.1007/s12272-009-1916-4 19784587

[B22] Ouyang S Sun LS Guo SL Liu X Xu JP Effects of timosaponins on learning and memory abilities of rats with dementia induced by lateral cerebral ventricular injection of amyloid beta- peptide Di Yi Jun Yi Da Xue Xue Bao 2005 25 2 121 126 15698986

[B23] Cai F Sun L Gao S Yang Y Yang Q Chen W A rapid and sensitive liquid chromatography-tandem mass spectrometric method for the determination of timosaponin B-II in blood plasma and a study of the pharmacokinetics of saponin in the rat J Pharm Biomed Anal 2008 48 5 1411 1416 10.1016/j.jpba.2008.09.032 19027255

[B24] Lu WQ Qiu Y Li TJ Tao X Sun LN Chen WS Antiplatelet and antithrombotic activities of timosaponin B-II, an extract of Anemarrhena asphodeloides Clin Exp Pharmacol Physiol 2011 38 7 430 434 10.1111/j.1440-1681.2011.05530.x 21517935

[B25] Forstl H Kurz A Clinical features of Alzheimer's disease Eur Arch Psychiatry Clin Neurosci 1999 249 6 288 290 10.1007/s004060050101 10653284

[B26] Backman L Jones S Berger AK Laukka EJ Small BJ Multiple cognitive deficits during the transition to Alzheimer's disease J Intern Med 2004 256 3 195 204 10.1111/j.1365-2796.2004.01386.x 15324363

[B27] Frost S Martins RN Kanagasingam Y Ocular biomarkers for early detection of Alzheimer's disease J Alzheimers Dis 2010 22 1 1 16 20847434 10.3233/JAD-2010-100819

[B28] Koronyo-Hamaoui M Koronyo Y Ljubimov AV Miller CA Ko MK Black KL Schwartz M Farkas DL Identification of amyloid plaques in retinas from Alzheimer's patients and noninvasive in vivo optical imaging of retinal plaques in a mouse model NeuroImage 2011 54 Suppl 1 S204 S217 20550967 10.1016/j.neuroimage.2010.06.020 PMC2991559

[B29] Chiu K Chan TF Wu A Leung IY So KF Chang RC Neurodegeneration of the retina in mouse models of Alzheimer's disease: what can we learn from the retina? Age (Dordr) 2012 34 3 633 649 10.1007/s11357-011-9260-2 21559868 PMC3337933

[B30] Nunomura A Perry G Aliev G Hirai K Takeda A Balraj EK Jones PK Ghanbari H Wataya T Shimohama S Oxidative damage is the earliest event in Alzheimer disease J Neuropathol Exp Neurol 2001 60 8 759 767 11487050 10.1093/jnen/60.8.759

[B31] Reed TT Pierce WM Markesbery WR Butterfield DA Proteomic identification of HNE-bound proteins in early Alzheimer disease: Insights into the role of lipid peroxidation in the progression of AD Brain Res 2009 1274 66 76 19374891 10.1016/j.brainres.2009.04.009

[B32] Smith MA Zhu X Tabaton M Liu G McKeel DW Jr Cohen ML Wang X Siedlak SL Dwyer BE Hayashi T Increased iron and free radical generation in preclinical Alzheimer disease and mild cognitive impairment J Alzheimer's Disease: JAD 2010 19 1 363 372 10.3233/JAD-2010-1239 PMC2842004 20061651

[B33] Lee HP Pancholi N Esposito L Previll LA Wang X Zhu X Smith MA Lee HG Early induction of oxidative stress in mouse model of Alzheimer disease with reduced mitochondrial superoxide dismutase activity PLoS One 2012 7 1 e28033 10.1371/journal.pone.0028033 22276093 PMC3261865

[B34] Butterfield DA Sultana R Methionine-35 of abeta(1–42): importance for oxidative stress in Alzheimer disease J Amino Acids 2011 2011 198430 22312456 10.4061/2011/198430 PMC3268025

[B35] Ma B Xu Q Zhao Y Xiong C Tan D Use of timosaponin Bll in the preparation of a medicament or product for the prevention and treatment of stroke Google Patents 2011

[B36] Yun D Bai-ping M Yu-wen C Yu-xian S Jing-jing Z Yu-jun S Protective effects of timosaponin BII on primary neurons against beta amyloid peptide 25–35 Chin Pharmacol Bull 2009 25 2 244 247

